# Advances in Sensing Techniques and Deep Learning for Food Quality Detection: Opportunities, Challenges, and Perspectives from Image Recognition to Multimodal Data Fusion

**DOI:** 10.3390/foods15183273

**Published:** 2026-09-16

**Authors:** Weihao Wang, Zhidan Jiang, Sisi Yang, Zhou Qin, Yongqiang Shi, Yaodi Zhu, Zhiyuan Sun

**Affiliations:** 1 Agricultural Product Processing and Storage Lab, School of Food Science and Engineering, Jiangsu University, Zhenjiang 212013, China; 2 Henan Institute of Food and Salt Industry Inspection Technology, Zhengzhou 450003, China; 3 College of Food Science and Technology, Henan Agricultural University, Zhengzhou 450002, China

**Keywords:** deep learning, food quality detection, image recognition, multimodal data fusion, model optimization

## Abstract

Food quality and safety inspection increasingly requires advanced solutions because conventional methods are challenged by the complexity of modern food systems. Effective inspection must address product quality, authenticity, and safety, together with process-related risks arising during production, storage, transportation, and distribution. However, existing analytical approaches and deep learning studies often focus on individual food attributes or sensing modalities, providing limited guidance for selecting appropriate models and sensing strategies for specific inspection objectives. This review systematically compares CNNs, RNNs/LSTMs, Transformers, GNNs, GANs, and hybrid architectures, as well as transfer learning, self-supervised learning, contrastive learning, few-shot learning, lightweight networks, edge computing, and multimodal fusion. Beyond predictive performance, we evaluate dataset size and representativeness, sample- and batch-level validation, external validation, data-leakage risks, interpretability, computational requirements, and the maturity of food-specific evidence. The principal contribution is an application-oriented framework linking inspection objectives and food matrices with sensing modalities, model architectures, validation evidence, and deployment conditions. Although Transformer-, GNN-, GAN-, multimodal-, and few-shot-learning-based approaches show substantial potential, many remain at developing, emerging, or prototype stages in food-specific applications. For high-risk targets, including toxicants, allergens, adulterants, and foodborne pathogens, deep learning systems should primarily support rapid screening and decision-making, while safety-critical results require confirmation using validated reference methods. Overall, this review provides a systematic perspective on deep learning for food quality and safety inspection and identifies key priorities for future research and industrial deployment.

## 1. Introduction

Food quality and safety represent one of the core concerns in modern society, directly impacting public health, social stability, and the sustainable development of the food industry [1]. Meanwhile, under the mounting pressure of global population growth, ensuring food safety and nutritional integrity poses a significant challenge to food production systems [2,3,4]. Furthermore, heightened consumer awareness of food quality drives the industry to pursue higher production efficiency, optimized quality control, and management approaches adaptable to complex supply chains. Regular monitoring of the entire food supply chain serves as the optimal solution to address quality and safety issues, enabling the detection of potential vulnerabilities and timely corrective actions.

Conventional food quality testing methods encompass sensory evaluation, physical measurements, instrumental analysis, and bioanalytical techniques. Sensory evaluation and physical measurements are commonly employed to assess attributes such as appearance, texture, firmness, and overall acceptability. Instrumental methods—including high-performance liquid chromatography (HPLC), ultra-high-performance liquid chromatography (UPLC), gas chromatography–mass spectrometry (GC-MS), liquid chromatography–mass spectrometry (LC-MS), and spectroscopic techniques—provide reliable and quantitative information on food composition, contaminants, authenticity markers, and quality-related chemical indicators [5,6]. Immunochemical assays, such as enzyme-linked immunosorbent assay (ELISA) and lateral flow immunoassay (LFIA), enable targeted detection of allergens, pathogens, toxins, and adulterants. Molecular genetic methods, including polymerase chain reaction (PCR), quantitative real-time PCR (qPCR), and loop-mediated isothermal amplification (LAMP), are widely applied for the identification of foodborne pathogens, genetically modified ingredients, and fraudulent substitutes.

Although these methods remain indispensable in most contexts, they exhibit significant practical limitations. Sensory evaluation is inherently subjective, with results highly susceptible to variations in the assessor’s expertise, fatigue levels, and environmental conditions, leading to poor stability [7]. Laboratory-based chemical and biological analytical methods, including chromatography and mass spectrometry, can provide accurate compositional data but typically require complex sampling and pretreatment procedures, costly instrumentation, and specialized personnel. Furthermore, mechanical measurements, though suitable for specific physical attributes, are predominantly contact-based and parameter-specific, rendering them inadequate for continuous monitoring.

Concurrently, unimodal sensing technologies face substantial information bottlenecks. RGB imaging and computer vision systems excel in evaluating color, shape, texture, surface defects, and packaging integrity, yet they offer limited insights into internal composition and volatile compounds. Spectroscopy and hyperspectral imaging can acquire chemically relevant information; however, their measurements are prone to interference from light scattering, sample heterogeneity, and high data dimensionality, and they encounter challenges in cross-instrument and cross-product calibration transfer. Chemical sensors, such as electronic noses and tongues, are sensitive to volatile or chemical properties, but their robustness is often compromised by sensor drift, cross-sensitivity, and environmental interference. The application of electronic nose technology in industrial production remains quite limited. This may be due to the challenges faced by current sensor technologies in terms of selectivity, repeatability, and reliability [8]. Modern food analytical methodologies emphasize rapid and precise techniques to ensure product quality, safety, authenticity, and labeling compliance [9]. Consequently, there is an urgent need for novel detection technologies and solutions within the modern food industry to further enhance detection efficiency and accuracy.

Deep learning is a subset of machine learning techniques that utilize multi-layered neural networks to extract complex feature representations with multi-level abstractions [10]. As an important branch in the field of artificial intelligence, deep learning has made remarkable progress in many fields, such as image recognition, speech recognition, and natural language processing, showing strong feature extraction and model generalization capabilities [11]. Its introduction in the field of food quality testing provides new ideas and tools to break through the limitations of traditional methods. Convolutional neural networks (CNNs), widely used in image processing, can automatically identify visual attributes of food products, including color, shape, and texture [10,12]. This image recognition-based detection method is not only objective and efficient, but can also achieve automation and real-time monitoring, which significantly improves the detection efficiency and accuracy. By harnessing the robust capabilities of deep learning technology, food quality inspection can shift from conventional manual methods to automated and intelligent detection frameworks. This transformation enhances both the efficiency and accuracy of inspection processes, enabling better alignment with the stringent demands of consumers regarding food quality standards.

Although image recognition technology has made significant progress in food quality testing, it is often difficult for single-modal data to fully reflect the complexity of food quality. Pioneering food authentication systems employed conventional machine learning algorithms, utilizing handcrafted feature extraction pipelines to isolate image characteristics [13]. The appearance of a food product may not fully reveal its internal composition or chemical properties. Therefore, multimodal data fusion technology came into being. Multimodal data fusion can more comprehensively reflect the quality characteristics of food by integrating multiple modal information, such as images [14], spectra [15], chemical indicators [16], electronic nose [17] and tongue [18], and IoT sensor data [19]. Multimodal data provide models with more comprehensive information, enabling them to make more rational decisions. Multimodal models have been proven to outperform single-modality models across various tasks [20]. This paradigm innovation not only improves the accuracy and reliability of detection but also provides a broader application prospect for food quality testing.

A search conducted in the Web of Science database on 1 July 2026, using keywords including “deep learning technology,” “food quality detection,” “food safety,” and “food authenticity,” yielded 2399 publications. The results were further refined by incorporating topic terms, such as “hyperspectral imaging,” “spectroscopy,” “electronic nose,” and “multimodal data fusion,” ultimately resulting in 374 articles included in this systematic review. Several existing reviews have summarized the fundamental applications of deep learning in food quality detection, encompassing food image recognition, defect detection, and spectral data analysis. However, few reviews have systematically addressed the recent evolution of deep learning from single-modality image recognition toward hyperspectral imaging, spectroscopy, electronic nose, electronic tongue, and multimodal data fusion. This review comprehensively summarizes the advances in deep learning for food quality detection, covering food appearance and defect recognition, composition prediction, authenticity and adulteration identification, contaminant and foodborne pathogen detection, as well as monitoring of spoilage, freshness, and processing and storage processes. Particular emphasis is placed on the application of CNNs, RNNs, and Transformer architectures integrated with hyperspectral imaging, spectroscopy, electronic nose, and IoT sensing technologies in food quality and safety assessment. Finally, this review discusses key challenges—including limited data availability, insufficient model interpretability, restricted cross-scenario generalization, and difficulties in industrial deployment—and outlines future prospects for deep learning in food quality detection.

## 2. The Technical Basis of Deep Learning and Food Quality Inspection

### 2.1. Deep Learning Methods and Algorithm Innovation for Food Inspection

#### 2.1.1. Convolutional Neural Networks (CNNs)

**Figure 1:**
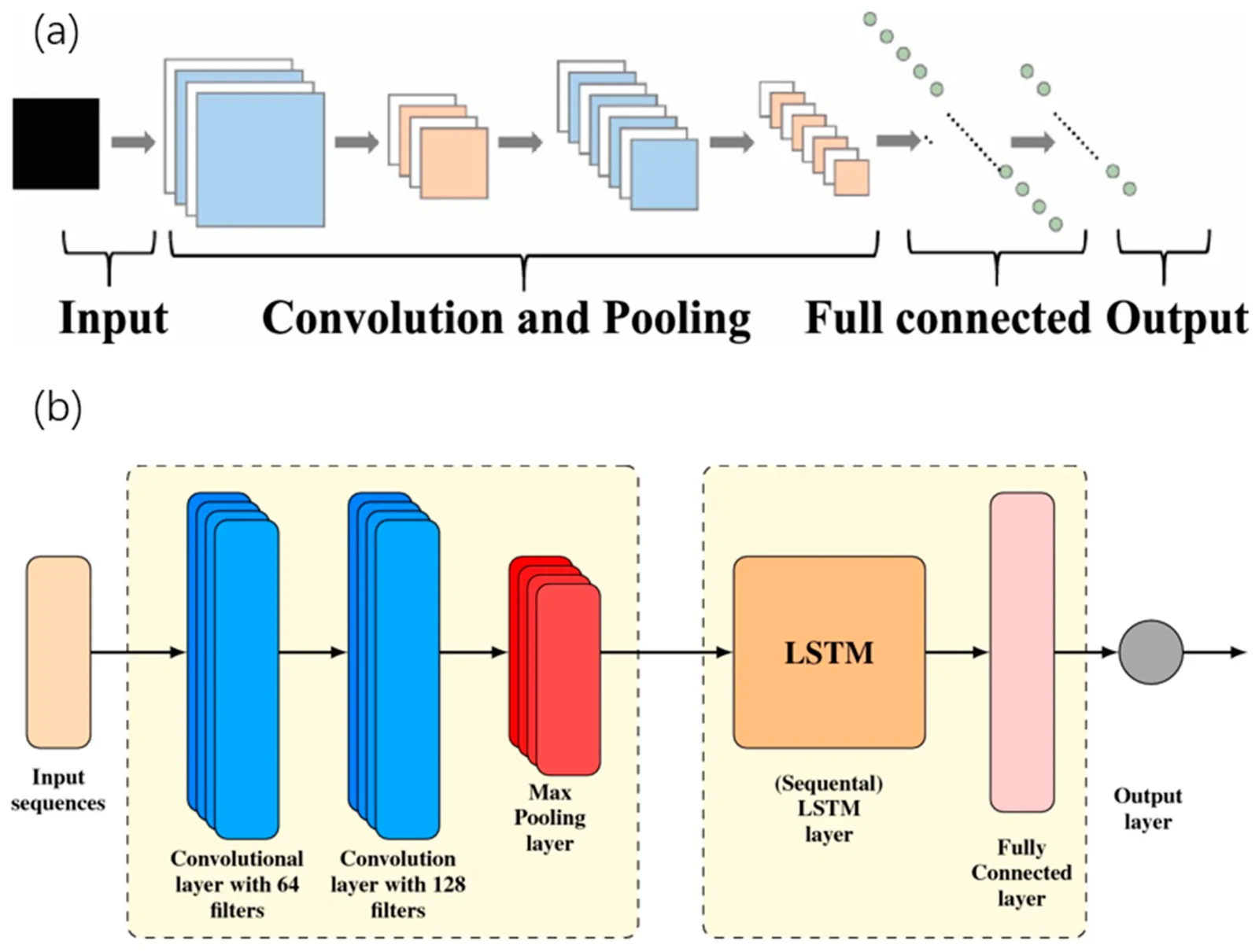
(**a**) The structure of a CNN. The figure was reproduced from [25], with permission from Elsevier, 2026. (**b**) A CNN–LSTM model for gold price time-series forecasting with two convolutional layers, a pooling layer, a LSTM layer, and an output layer. The figure was reproduced from [29], with permission from Springer Nature, 2020.

Convolutional neural networks (CNNs) are one of the most commonly used architectures in deep learning, widely applied in agriculture, industry, transportation, and other fields, especially in image recognition and processing [21,22,23]. As shown in Figure 1a, core components of CNNs include convolutional layers to detect local patterns, pooling layers to reduce spatial dimensions and enhance invariance, and fully connected layers for final classification or regression tasks [24,25]. The convolutional layers capture local features, the pooling layers conduct downsampling to reduce dimensionality, and the activation functions introduce non-linearity. The fully connected layers then convert the two-dimensional feature maps from the preceding layers into one-dimensional feature vectors. Through these processes, hierarchical features in images are effectively extracted, enabling the completion of various visual tasks.

The application of CNNs in food quality detection primarily focuses on the classification of digital images and the recognition of objects within images [26]. CNNs are particularly well suited for food quality assessment tasks involving spatially structured data, such as RGB, hyperspectral, microscopic, and packaging images. The primary advantage of CNNs lies not only in their ability to automatically extract image features but also in their utilization of local connectivity, shared convolutional kernels, and hierarchical representation learning to identify quality-related patterns across varying spatial scales. In food applications, shallow layers typically capture low-level features, such as color transitions, edges, and textures, whereas deeper layers learn more abstract representations associated with defects, contamination, shape irregularities, or product categorization. Ong et al. employed HSI combined with deep learning to detect the total aerobic colony count on eggshells, enabling safety assessment of eggshell surfaces [27]. Leveraging the powerful self-learning and feature extraction capabilities of CNNs, along with their progressive, layer-by-layer feature learning, they integrated a channel attention (CA) mechanism with depthwise separable convolution (DSC) to quantify the total aerobic plate count (APC) on eggshell surfaces. Across a dataset of 108 egg samples, the model achieved a calibration correlation coefficient (RC) of 0.9756 and a prediction correlation coefficient (RP) of 0.8959, with a root mean square error of calibration (RMSEC) of 0.1146 and a root mean square error of prediction (RMSEP) of 0.2396.

CNNs are also applicable to various forms of spectral and hyperspectral data. One-dimensional CNNs (1D-CNNs) are typically employed to extract local correlations along the wavelength dimension, whereas two-dimensional CNNs (2D-CNNs) focus on spatial patterns within spectral images. Three-dimensional CNNs (3D-CNNs) can jointly model spatial and spectral information; however, their increased parameter size may elevate the risk of overfitting and computational costs. In practice, CNNs are frequently integrated with chemometric methods or other deep learning architectures. For instance, Zeng et al. [28] developed a CNN regression model incorporating Savitzky-Golay smoothing and standard normal variate (SNV) transformation to predict the soluble solids content of apples based on full-spectrum diffuse reflectance. This model demonstrated robust predictive performance (*R_p_ =* *0.95, RMSEP = 0.59%*). These studies indicate that CNNs can effectively translate complex spectral-spatial information into discriminative representations. Nevertheless, their effectiveness is highly dependent on the quality of spectral calibration, wavelength selection, sample representativeness, and the consistency of the imaging instrumentation.

CNNs can also be integrated with models such as Long Short-Term Memory (LSTM) networks (Figure 1b) or employ different types of convolutional layer configurations to process time-series data or extract features from multi-dimensional data [29]. CNN–LSTM models integrate spatial feature extraction with temporal modeling, making them suitable for video streams, spectral sequences, or cold chain monitoring data. CNN–Transformer models complement local convolutional features by capturing global dependencies. Furthermore, CNN-based branches can be integrated with spectral, chemical, or sensor-specific networks for multimodal quality prediction.

In addition, variants of CNNs, such as ResNet (residual network) and Inception network, further improve the performance and generalization ability of the model by introducing residual joining and multi-scale feature extraction [23].

#### 2.1.2. Recurrent Neural Networks (RNNs) and Their Variants

Recurrent Neural Networks (RNNs) and their variants, such as Long Short-Term Memory (LSTM), have unique advantages in processing sequence data. In food quality testing, RNNs can process sequence data, obtain comprehensive values through weighting, and use comprehensive values for prediction and detection. These characteristics enable RNNs to be applied in the monitoring of food production and distribution processes. For example, in cold chain transportation, temperature and humidity sensors directly measure and record environmental conditions. RNNs do not replace these sensors; rather, they analyze the resulting time-series data. By learning temporal dependencies, RNN-based models can detect anomalous fluctuations, prolonged or recurring temperature deviations, and departures from expected transport profiles. RNNs can also incorporate transport duration and other sensor variables to support cumulative exposure assessment, risk prediction, and early warning.

**Figure 2:**
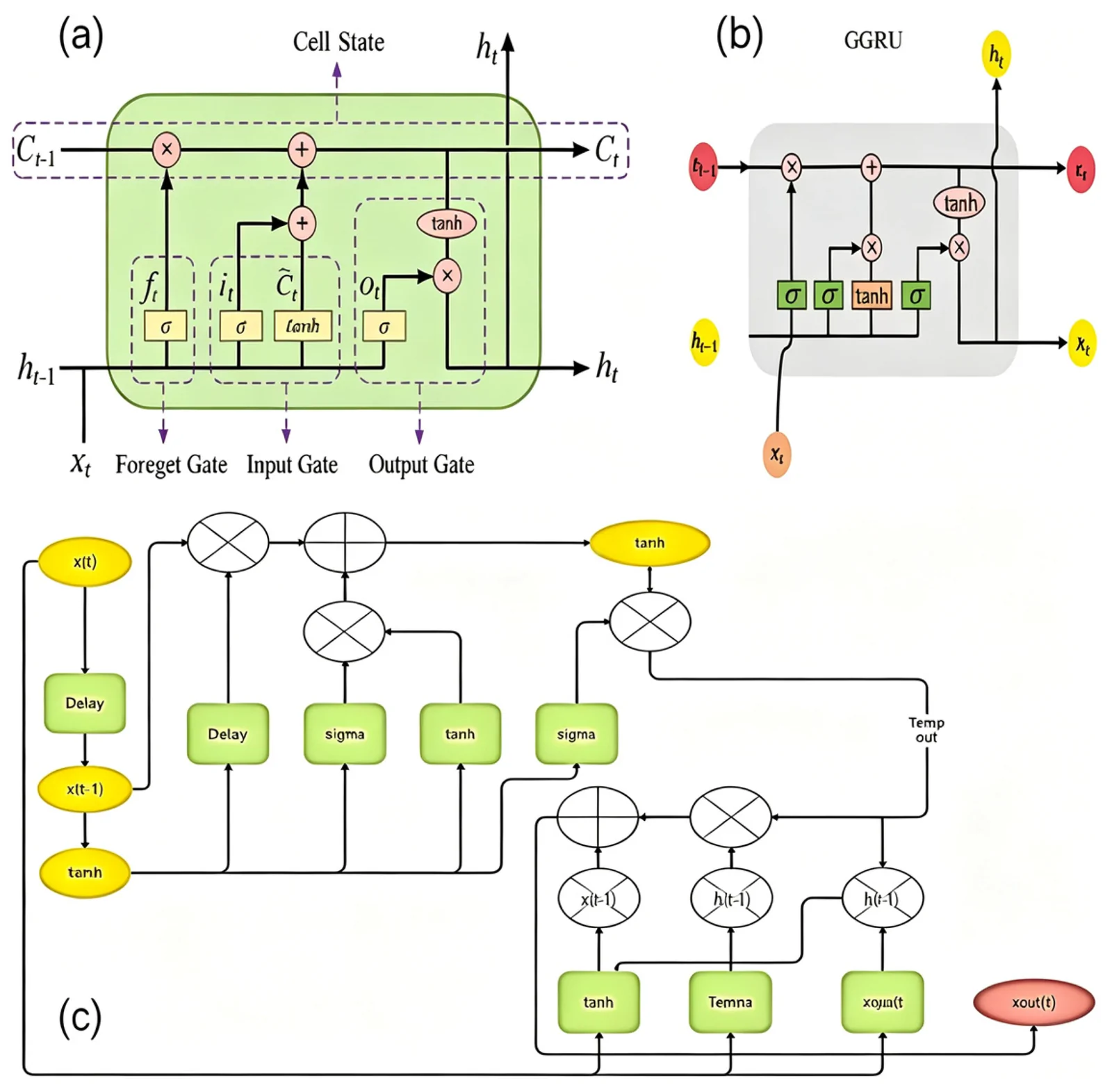
Some structure examples of the architecture of Long Short-Term Memory (LSTM) models. (**a**) The memory block of the LSTM. The figure was reproduced from [30], with permission from Elsevier, 2023. (**b**) The architecture of LSTM. The figure was reproduced from [25], with permission from Elsevier, 2026. (**c**) Design of the LSTM- and GRU-based model for extraction of multimodal feature sets. The figure was reproduced from [33], with permission of the Creative Commons CC-BY, 2025.

The LSTM is an improved architecture of the RNN and stands as one of the state-of-the-art RNN variants, offering advantages such as rapid convergence speed and high prediction accuracy. It has achieved success in fields like speech recognition, visual recognition, time-series model prediction, risk warning models, and particularly in the domain of video information recognition [30,31]. As shown in Figure 2a,b, in an LSTM network, the flow of information during learning is controlled by three internal gates: the forget gate, the input gate, and the output gate [25]. Through this gating mechanism, the LSTM can selectively retain and update information [32]. As shown in Figure 2c, Said et al. combined the feature extraction and selection techniques of LSTM and Gated Recurrent Unit (GRU), where the fusion of LSTM and GRU models generates a multi-scale feature set, enabling the model to capture both temporal and spatial features [33]. Leveraging its unique gating mechanism, LSTM offers significant advantages in processing sequential data with long-term dependencies and retaining information from distant time steps. It can dynamically regulate the flow of information, allowing the model to flexibly learn critical features while filtering out irrelevant noise [34]. In food inspection, this capability makes LSTM highly suitable for processing spectral data. By treating wavelengths as an ordered sequence, LSTM can capture chemical correlations between both adjacent and distant wavelengths, thereby learning sequential dependencies among spectral measurements to accurately predict food quality attributes or adulteration levels.

Meanwhile, a model combining CNN and LSTM possesses the capability to handle spatiotemporal data, such as analyzing images of food [35,36]. In the CNN–LSTM architecture, CNNs typically extract spatial or local spectral features from individual frames or measurements, while LSTMs model their temporal evolution or sequential dynamics. This design is particularly well suited for video-based inspection, dynamic production line monitoring, and sequential analysis of spectral or sensor measurements. In order to capture the correlational information among different spectral wavelengths, Xing et al. leveraged the ability of LSTM to retain effective sequential information and learn dependencies among ordered input variables [37]. They incorporated an LSTM module into a 1D-CNN–self-attention model and constructed a hybrid architecture to detect *Staphylococcus aureus* (*S. aureus*) in lamb meat. The model achieved optimal performance when built upon seven characteristic wavelengths selected by two-dimensional correlation spectroscopy (2D-COS), yielding a coefficient of determination (*R*^2^) of *0.8911*, a root mean square error (*RMSE*) of *0.6601*, and a ratio of performance to deviation (*RPD*) of *3.04* on the test set. These results demonstrate that the integration of 2D-COS with the 1D-CNN–LSTM–self-attention model enables effective detection of *S. aureus* concentration in lamb meat.

#### 2.1.3. Transformer Architecture

**Figure 3:**
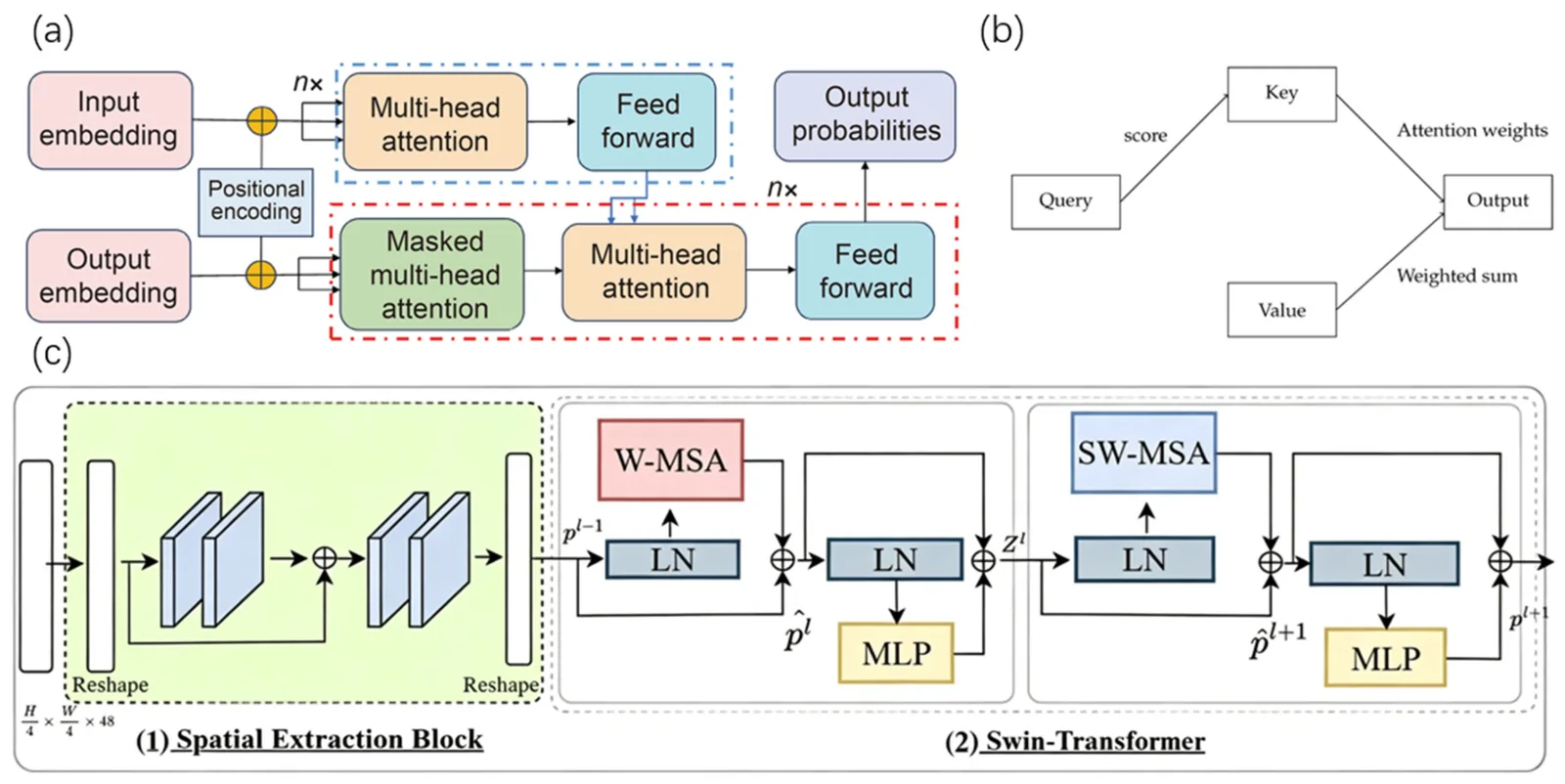
(**a**) Schematic diagram of Transformer model. The Transformer model consists of an encoder, decoder, self-attention mechanism, and feedforward neural networks. The figure was reproduced from [39], with permission of the Creative Commons CC-BY, 2025. (**b**) Schematic representation of the attention mechanism. The figure was reproduced from [41], with permission of the Creative Commons CC-BY, 2023. (**c**) The architecture of Swin Transformer with spatial extraction. (**1**) The structure of the spatial extraction block. (**2**) The structure of the Swin-Transformer module. The figure was reproduced from [45], with permission from Springer Nature, 2023.

Originally designed for natural language processing tasks, the introduction of the Transformer model has become a breakthrough in various fields, particularly excelling at processing image feature information [38]. As shown in Figure 3a, the Transformer model consists of an encoder, a decoder, a self-attention mechanism, and a feedforward neural network [39]. The Transformer architecture excels at leveraging the attention mechanism, as illustrated in Figure 3b, to capture contextual information and long-range dependencies, making it highly effective for image understanding and text segmentation tasks [40,41]. Therefore, detection methods based on multi-dimensional feature extraction networks and Transformer models are highly suitable for food quality assessment [42]. By employing a shifted window attention mechanism, the Transformer architecture can automatically learn cross-modal correlations and effectively capture feature relationships across different regions, thereby achieving more accurate assessments [43]. In food quality assessment, accurate food image recognition and classification are essential for addressing food-related challenges. Among these, fine-grained visual classification of food aims to distinguish subcategories within the same parent category. Since different subcategory instances typically exhibit only subtle differences, it is necessary to compare both local and global features of different subcategory objects in fine detail [44]. Transformers are particularly well suited for such fine-grained discrimination tasks. For example, Kim et al. fuses the Transformer technology with four modules [44], Local Feature Extraction Network (L-FEN), Convolutional Patch Merging (CP), Multipath (MP), and Multi-View (MV), to solve fine-grained food classification tasks, and achieves *66.75%*, *85.78%*, and *92.93%* accuracy on the three main food datasets of ISIA Food-500, UEC Food-256, and Food-101. Based on the Swin Transformer method, Lee et al. proposed a new multi-food detection method [45], which utilizes an improved Swin Transformer and Recursive Feature Pyramid Network (MFD-MST) and a Swin Transformer with a spatial extraction block (STSE) as the skeleton to identify items in multi-food images to improve the local and structural information of the image (Figure 3c) [45]. Its model MFD-MST outperformed the Swin Transformer on the three food datasets, respectively.

Transformers typically exhibit a weak inductive bias toward local features, often requiring large-scale datasets or effective pretraining to achieve stable performance [46]. This poses a significant challenge in food quality research, where datasets are usually limited in size and may suffer from class imbalance, batch effects, and variations arising from differences in food cultivars, imaging equipment, illumination conditions, and production environments. Under insufficient training data, Transformer models are prone to overfitting and may fail to generalize to samples acquired under novel conditions. Therefore, CNNs and Transformers should be regarded as complementary rather than competing architectures. CNNs maintain advantages in computational efficiency, local texture extraction, and deployment on resource-constrained devices, whereas Transformers excel in capturing global context and long-range feature dependencies [47]. Consequently, hybrid CNN–Transformer architectures are often more suitable for food inspection tasks that necessitate simultaneous local defect identification and global quality assessment.

#### 2.1.4. Fusion Based on Graph Neural Networks

**Figure 4:**
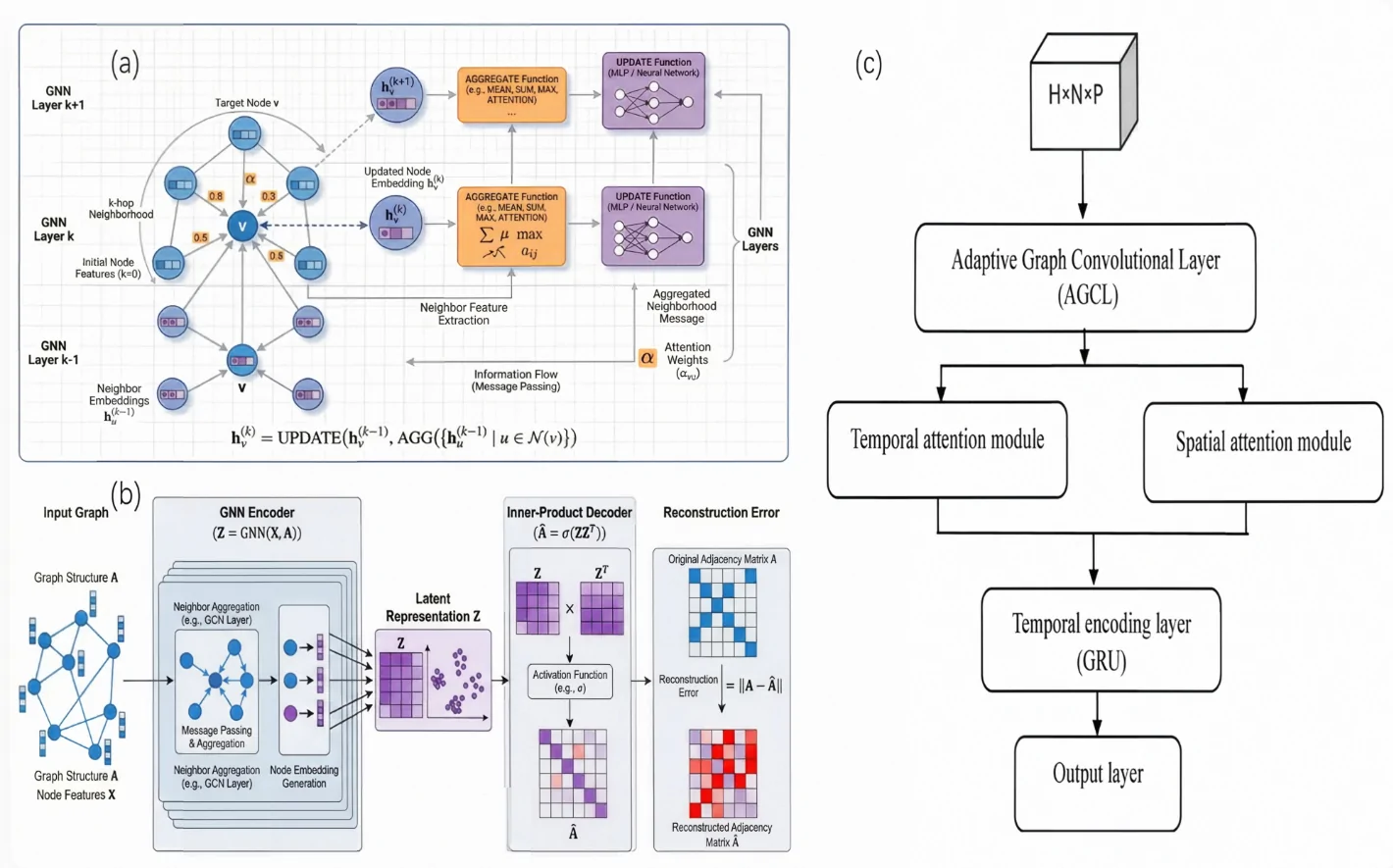
(**a**) Conceptual architecture of neighborhood aggregation in GNNs. The figure was reproduced from [50], with permission of the Creative Commons CC-BY, 2026. (**b**) GAE architecture. The figure was reproduced from [50], with permission of the Creative Commons CC-BY, 2026. (**c**) Structure of E-STGNN. The figure was reproduced from [51], with permission from Elsevier, 2025.

Graph neural networks (GNNs) have attracted a lot of attention for their effectiveness in processing and learning graph-structured data [48]. GNNs can construct label graphs based on the correlation between labels and perform feature extraction and representation learning on the graphs. As shown in Figure 4a, GNNs consist of graph convolutional layers that aggregate hidden node representations by gathering feature information from node neighborhoods [49,50]. This information passes through two key parts: pooling and Readout layers, which reduce graph scale and aggregate node information to transition from node-level features to graph-level representations. Furthermore, variants of GNNs, such as GAEs (Figure 4b) and STGNNs (Figure 4c), possess capabilities like graph reconstruction and capturing spatial dependencies and temporal evolution [50,51].

GNNs have been applied to social networks, recommender systems, chemical molecule prediction, physical systems, knowledge graphs, and other fields [52]. In food quality assessment, graph nodes can be defined at multiple levels. At the feature level, nodes may represent wavelength bands in hyperspectral data, volatile compounds detected by electronic noses, physicochemical indicators, or image regions extracted via CNNs. Edges can reflect spectral adjacency, correlation coefficients, chemical interactions, spatial proximity, or learned similarity relationships. For instance, Yang et al. [53] reconstructed spectral sequences into graph-structured data and proposed a Graph Isomorphism Network augmented with a Node-level Scalar Attention mechanism (NSA-GIN). This framework effectively encodes latent dependencies among diverse spectral representations and accurately maps them to food compositional contents, achieving *R*^2^ values of *0.9011*, *0.9529*, and *0.9564* across three datasets. Concurrently, GNNs have demonstrated significant potential in food safety risk prediction and contamination monitoring. Food contamination is typically influenced by multiple interrelated factors, including raw material origins, processing environments, storage conditions, transportation routes, hygiene protocols, and environmental variables. Graph representations can explicitly model these dependencies, thereby facilitating risk propagation analysis [54].

In the food industry, data of different modalities can be represented as graph structures, and graph attention networks (GATs) can be used to dynamically measure the relationships between different modalities, so as to focus on related parts more precisely. Tran-Anh and Vu leveraged image and text data for comprehensive feature extraction, and the extracted features were fused and processed using GATs to capture complex relationships in multimodal data [55]. Meanwhile, leveraging the feature correlation capabilities of GNNs, food quality contamination assessment is another area where GNNs are applied. Yan et al. incorporated contrastive learning into a GNN model, and their developed CSGNN model achieved an AUC and recall rate of *0.9188* and *1.0000*, respectively [56]. This feature extraction capability enables GNNs to integrate features and relationships from different types of data in multimodal fusion, thereby yielding more accurate and comprehensive results.

#### 2.1.5. Generative Adversarial Networks (GANs)

Generative Adversarial Networks (GANs) comprise a generator and a discriminator, which engage in an adversarial training process to compete and learn from each other. In the field of food quality inspection, a critical application of GANs is data augmentation, particularly when training data are scarce. This is attributed to the widespread use of CNNs in the food sector, where their development relies on large-scale databases for training. Processing data with GANs enables neural network models to effectively learn intra-class features and inter-class distinctions, thereby enhancing robustness and mitigating overfitting. For example, to enable rapid and non-destructive detection of Listeria in fresh cheese, Meenakshi et al. combined near-infrared spectroscopy with both machine learning (random forest (RF) and support vector machine (SVM)) and deep learning (1D-CNN) approaches [57]. Meanwhile, to augment the spectral dataset, they employed a conditional Wasserstein GAN with gradient penalty (cWGAN-GP) for spectral augmentation. The spectral augmentation improved the performance of the 1D-CNN, achieving a classification accuracy of *95.6%*, and inference time was also improved (*0.13–0.14* s) compared to using real spectra.

Furthermore, GANs can incorporate class labels, environmental conditions, or quality attributes into the generation process, enabling the synthesis of samples with specified characteristics. This conditional design is more suitable for food inspection than unconditional generation, as it allows for the targeted simulation of specific defect types, illumination conditions, food categories, or processing stages [58]. For example, Navarro et al. introduced a conditional GAN (cGAN) model for grape berry segmentation in computer vision, attaining an accuracy of *0.9970*, precision of *0.9862*, recall of *1.0000*, F1-score of *0.9930*, and IoU of *0.9813* during segmentation [59].

However, GAN-based image processing is not without limitations. Its approach to handling spectral data often results in excessive smoothing, leading to the loss of fine textural details in images [56]. Furthermore, the training process of GANs is inherently unstable, frequently manifesting as mode collapse and vanishing gradients. Meanwhile, the generated samples may fail to faithfully represent rare or complex defects, particularly when the original training set does not adequately capture the underlying variability. Overall, GANs are most valuable when the primary challenges involve data scarcity, class imbalance, or controlled image generation. They are less suitable as standalone decision-making models, nor can they independently address issues arising from poor measurement quality, insufficient annotation, or distribution shift. Table 1 summarizes the fundamental principles, application advantages, and limitations of five kinds of deep learning approaches. Each methodology demonstrates distinct technical characteristics that require systematic evaluation to optimize their synergistic implementation in practical food inspection frameworks.

### 2.2. Algorithm Innovation

#### 2.2.1. Lightweight Model Design, Edge Computing, and Transfer Learning

As multiple deep learning technologies have been applied in food quality inspection, some drawbacks in their use have gradually emerged. This has also prompted the adoption of new methods within the field of food quality detection.

Currently, the application of deep learning models in food quality detection faces dual challenges of computational resource limitations and real-time performance requirements. Due to their high computational demands and lengthy processing times, deep learning models struggle to be deployed on mobile platforms, thus making them unsuitable for rapid, real-time food detection [60].

Future research will place greater emphasis on the design of lightweight models to meet the demands of edge computing. Lightweight models reduce computational requirements through efficient network architectures, model compression, knowledge distillation, quantization, and hardware-aware optimization. Among these strategies, lightweight network design represents the most direct approach, with representative architectures including MobileNet and YOLOv4. Techniques such as pruning (removing redundant weights), quantization (reducing numerical precision), knowledge distillation (training compact models to mimic larger ones), and neural architecture search (NAS) have been employed to optimize models for edge environments [61]. These techniques are essential for deploying models in low-power applications.

For example, lightweight model architectures such as MobileNet and EfficientNet significantly improve the operational efficiency of models by reducing the number of model parameters and the amount of computation. These lightweight models reduce floating-point operations (FLOPs), decrease processing time, and minimize model size [62]. To address the challenges of high hardware requirements in deep neural network models, Ji et al. utilized a lightweight EfficientNet feature network to optimize the YOLOv4 model [63], reducing the number of model parameters. While the accuracy of the enhanced YOLOv4 decreased by approximately *2*%, its inference speed improved significantly by *43*%. Teng et al. integrated four lightweight CNN models into 3D-printed labels and tested them on three types of fruit: kiwifruit, green mango, and persimmon. Among them, the MobileNet model achieved a prediction accuracy of *93*%. Lightweight CNN models can extract features from label images and build predictive models to assess fruit freshness from the images [64]. Overall, lightweight deep learning models provide a crucial foundation for edge-based food quality inspection. Their primary advantage lies not merely in reduced architectural size, but in achieving an appropriate balance among detection performance, computational efficiency, energy consumption, and deployment cost. In addition, the rapid development of edge computing technology provides new opportunities for the application of lightweight models.

Edge computing aims to achieve lower latency, reduce communication overhead, enhance privacy protection, and provide more stable on-site operation by relocating computation and inference closer to the data source, thereby enabling real-time food quality assessment under resource-constrained conditions. To make edge computing viable for food inspection in such environments, deep learning models must also be designed with lightweight architectures to facilitate efficient on-device inference [65].

The value of edge computing is particularly evident in cold chain logistics and warehouse monitoring. Food spoilage may be influenced by time-varying factors, such as temperature, relative humidity, gas composition, vibration, storage duration, and transportation conditions. Unlike the relatively stable conditions on production lines, cold chain transportation is typically characterized by high mobility, limited energy supply, and unreliable network connectivity [65]. Edge gateways can leverage lightweight temporal models, anomaly detection algorithms, or hybrid rule-model systems to locally acquire and process these sensor data streams. Such local processing enables early warnings when abnormal refrigeration conditions, packaging damage, or potential quality deterioration are detected, without the need to continuously transmit all raw sensor data. This is especially critical for refrigerated vehicles, warehouses, retail storage facilities, and remote production sites, where communication quality may fluctuate.

Transfer learning (TL) is also an important method to improve generalization ability. By pretraining models on large-scale general datasets and subsequently transferring and fine-tuning them for specific food quality detection tasks, the adaptability of the models to novel tasks can be significantly enhanced [66]. Furthermore, pretraining facilitates improved performance even under few-shot learning conditions [67,68]. Razavi et al. applied the transfer technique to the Retset model [69]. ResNet is good at learning images and, by combining residual learning and well-structured architecture, and optimizing the generalization ability of the model by transfer learning technology, the model showed good performance in rice identification and detection, and successfully identified six rice varieties with an accuracy of more than *99.85*%. Transfer learning utilizes the weights and biases of a network trained on one task to enhance performance in new applications. Such fine-tuning practices are not uncommon in food industry inspection and supply chains, which is why transfer learning generally achieves favorable results in food detection.

#### 2.2.2. Self-Supervised Learning and Unsupervised Learning

Self-supervised learning and unsupervised learning are the current research hotspots in the field of deep learning, and their applications in food quality testing have broad prospects. Self-supervised learning can significantly reduce the reliance on large-scale labeled data by leveraging unlabeled data for pretraining [70]. For example, self-supervised temporal learning (STL) networks and adaptive learning units (ALUs) construct a self-supervised task by performing an erasure and reconstruction process [71]. Furthermore, the K-nearest neighbor (KNN) model, as a supervised learning algorithm, determines the category of a target sample by adhering to the majority principle—classifying the sample based on the most frequent category among its K-nearest neighboring samples [72]. Self-supervised learning is also widely used in spectral analysis, enabling models to extract informative features directly from raw spectral data during the pretraining phase, thereby reducing reliance on manual preprocessing and improving data analysis efficiency. In addition to enhancing preprocessing efficiency, self-supervised learning can also be applied during the fine-tuning stage to refine models with limited labeled data, significantly improving training efficiency and generalization capability. Zhou et al. integrated hyperspectral imaging with self-supervised learning to develop an adaptive spectral feature regression network for zinc content prediction, achieving high detection accuracy (*Rp*^2^ *= 0.97, RMSEP = 8* mg/kg, *RPD = 6*) [73]. Self-supervised and unsupervised learning leverage attention mechanisms to assist models in learning and discriminating targets, thereby enhancing representational performance. Their contrastive learning capability is widely applied in the visual domain. In the food industry, this ability not only improves model performance but also demonstrates value in food classification and spectral data analysis.

Unsupervised learning uses clustering, dimensionality reduction, and other technologies to automatically discover hidden structures in data. For example, Ördek et al. employed an enhanced unsupervised learning algorithm to identify required parts [74]. This algorithm automatically matches components suitable for additive manufacturing by recognizing their geometric features, thereby streamlining preliminary preparations for the manufacturing process. In the field of food inspection, clustering and dimensionality reduction techniques can also be applied to anomaly detection: by analyzing the distribution characteristics of normal-quality samples, samples that deviate from this distribution can be identified. Depending on the data type, the detected anomalies may include surface defects, foreign substances, spoilage, spectral anomalies associated with contamination or adulteration, and abnormal temperature or humidity conditions during storage and transportation. This detection approach is particularly valuable when anomalous samples are rare or difficult to characterize precisely.

#### 2.2.3. Model Interpretation Tools and Data Protection

Most of the current deep learning models belong to the “black box” model, and their internal decision-making process is difficult to explain. The “black box” nature of deep learning models may undermine trust and make it difficult to assign responsibility when erroneous decisions occur. In general, these models are better suited for low- and medium-risk tasks, such as preliminary screening, appearance assessment, process monitoring, and sample prioritization. For decisions involving high risk or regulatory compliance—such as final product release or confirmation of contamination or adulteration—reliance should not be placed solely on deep learning predictions. Instead, such decisions should be subject to human review and verified using established analytical methods. To ensure clear accountability, food inspection systems should document all input data, model versions, prediction results, uncertainty factors, operator review records, and final decisions. Explainable artificial intelligence (XAI) can provide interpretable evidence for predictions generated by deep learning models, thereby enhancing the transparency and acceptability of intelligent food quality inspection systems [75,76]. By identifying relevant image regions, spectral bands, chemical indicators, or sensor variables, XAI methods enable users to determine whether predictions are grounded in meaningful food quality features or irrelevant background information. Common approaches include feature importance analysis, saliency maps, Local Interpretable Model-agnostic Explanations (LIME), and SHapley Additive exPlanations (SHAP). In the context of food inspection, these methods can assist researchers in identifying defect regions, characteristic spectral wavelengths, or anomalous sensor patterns associated with deterioration, contamination, or adulteration. Through the identification and analysis of such indicators, XAI facilitates model validation and error analysis.

With the widespread application of image recognition technology, data security and privacy protection have become a major challenge. In food quality testing, a large amount of sensitive information is involved, and how to ensure data security is key. First and foremost, data encryption technology can ensure the security of data during data transmission and storage. For example, the Okamoto Uchiyama encryption algorithm was used to strengthen the privacy and security of IoT-generated data [77], protecting the data privacy and security of a lightweight blockchain and IoT integrated system. Furthermore, differential privacy protects individual privacy and preserves sensitive data by adding specific noise values, thereby enhancing the privacy of the data [78]. Additionally, federated learning can enhance data transmission performance through distributed machine learning, reducing both data transfer and logistical complexity, while blockchain and distributed ledger technologies can provide deep learning systems with reliable capabilities for data recording, sharing, and traceability. These methods safeguard the security and privacy of data during transmission and storage in food quality inspection, while ensuring traceability and verifiability of data sources.

### 2.3. Multimodal Data Characteristics

#### 2.3.1. Multimodal Data and Its Processing

Multimodal data refers to collections derived from multiple sources or formats. Integrating information from different modalities provides a more comprehensive perspective, significantly enhancing the understanding and analytical capabilities for complex phenomena [79]. In the field of food inspection, the fusion of multimodal data offers robust support for safety and quality control. For instance, chemical sensor data are utilized for precise, real-time detection of harmful substances, substantially improving the reliability of safety monitoring [80], image data are used to assess the visual quality of food, near-infrared (NIR) spectroscopy data enable rapid analysis of food components [81], and volatile compound information captured by electronic nose sensors can be integrated with chemical sensor data and spectral analysis for more comprehensive evaluation [82], significantly enhancing result reliability and robustness. Effective fusion of multimodal data relies on preprocessing and feature extraction. Preprocessing aims to eliminate noise, simplify data, and enhance features. Common methods include normalization, cropping, and rotation of images to increase diversity and robustness, baseline correction and smoothing of spectral data to remove noise and artifacts [83], and standardization of chemical indicator and IoT sensor data to eliminate dimensional effects [84]. Feature extraction is the critical step of mapping data from different modalities into a common feature space. CNNs can automatically extract local and global features from images, while methods such as Principal Component Analysis (PCA) and Linear Discriminant Analysis (LDA) are commonly employed to extract key features from spectral data.

#### 2.3.2. Technical Roadmap of Multimodal Data Fusion

The overall technical workflow for multimodal food quality inspection should encompass five sequential stages: multimodal data acquisition, data synchronization and preprocessing, feature extraction for each modality, multimodal data fusion, and final quality and reliability assessment. During the data acquisition stage, images, spectral data, chemical indicators, sensory attributes, and IoT monitoring data must be collected from the same sample or supply chain node. Since these data may differ in spatial resolution, sampling frequency, dimensionality, and measurement scale, data synchronization, standardization, calibration, and handling of missing values are required prior to data fusion.

The complementarity among various detection modalities constitutes the primary rationale for multimodal data fusion in food quality inspection. Image data are primarily used to characterize external morphology and surface defects, whereas spectral and chemical data provide information on internal composition and various quality or safety indicators. Electronic noses and electronic tongues can capture the overall profiles of odor and taste. Furthermore, IoT sensors can record environmental and time-dependent data throughout processing, storage, and transportation. By integrating these data, a comprehensive understanding of the appearance, internal composition, sensory characteristics, and environmental conditions of the product can be achieved, thereby overcoming the information gaps inherent in single-modality approaches. For example, Nargesi et al. integrated spectroscopic and imaging technologies to simultaneously capture spectral and visual information of food products, and combined these with machine learning algorithms to detect adulteration levels in red chili powder, achieving accuracies of *98.8*%, *100*%, and *97.8*% for adulterants of chickpea flour, rice husk powder, and wheat flour, respectively [85]. In crop inspection, Guo et al. employed hyperspectral imaging to combine spectral and image data for the detection of mycotoxins in cereals [86]. These approaches integrate spectrally derived chemical composition information with spatial information related to the location and morphology of contamination. Such integration is complementary for addressing food adulteration and contamination detection, as spectral features can indicate chemical or biochemical changes, while spatial information enables the localization of heterogeneous contamination, thereby improving detection accuracy.

Depending on the timing of information integration, multimodal fusion can be categorized into early fusion, intermediate fusion, and late fusion. Early fusion involves directly concatenating standardized data from different modalities prior to model training. Intermediate fusion first extracts modality-specific feature representations and then integrates them through concatenation, attention mechanisms, gating mechanisms, or similar strategies. Late fusion allows each modality to generate predictions independently and then combines the individual predictions through weighting, voting, stacking, or other aggregation methods. In practical food inspection applications, when different modalities exhibit substantial differences in dimensionality, sampling frequency, or data quality, intermediate fusion and late fusion tend to offer greater flexibility.

## 3. Innovative Application of Image Recognition Technology

### 3.1. Food Appearance Quality Assessment

Image-based food inspection encompasses safety-related quality assessment as well as food identification, and these two aspects should be clearly distinguished. The former aims to detect defects, spoilage, contamination, adulteration, and variations in various measurable quality indicators in food products, while the latter primarily focuses on classifying food items into their respective categories or specific varieties based on their visual appearance [87].

The appearance of food is one of the important indicators for quality evaluation, including color, shape, texture, etc. Traditional visual quality assessment relies on artificial visual inspection, which is not only inefficient, but also susceptible to subjective factors. For this reason, colorimetric sensor [88], near-infrared spectroscopy [88], hyperspectral imaging [89], or computed imaging techniques [90] are often used to quantitatively characterize the color of food products [91]. At the same time, standalone computer vision technology is also susceptible to lighting conditions, which can be effectively addressed by integrating algorithms with spectral technology [92,93,94]. Furthermore, deep learning networks enable the recognition, segmentation, and localization of images, while possessing the capability to process large volumes of data, thereby effectively overcoming the subjectivity, slowness, and inaccuracy inherent in traditional visual quality assessment methods and machine learning algorithms [95,96].

**Figure 5:**
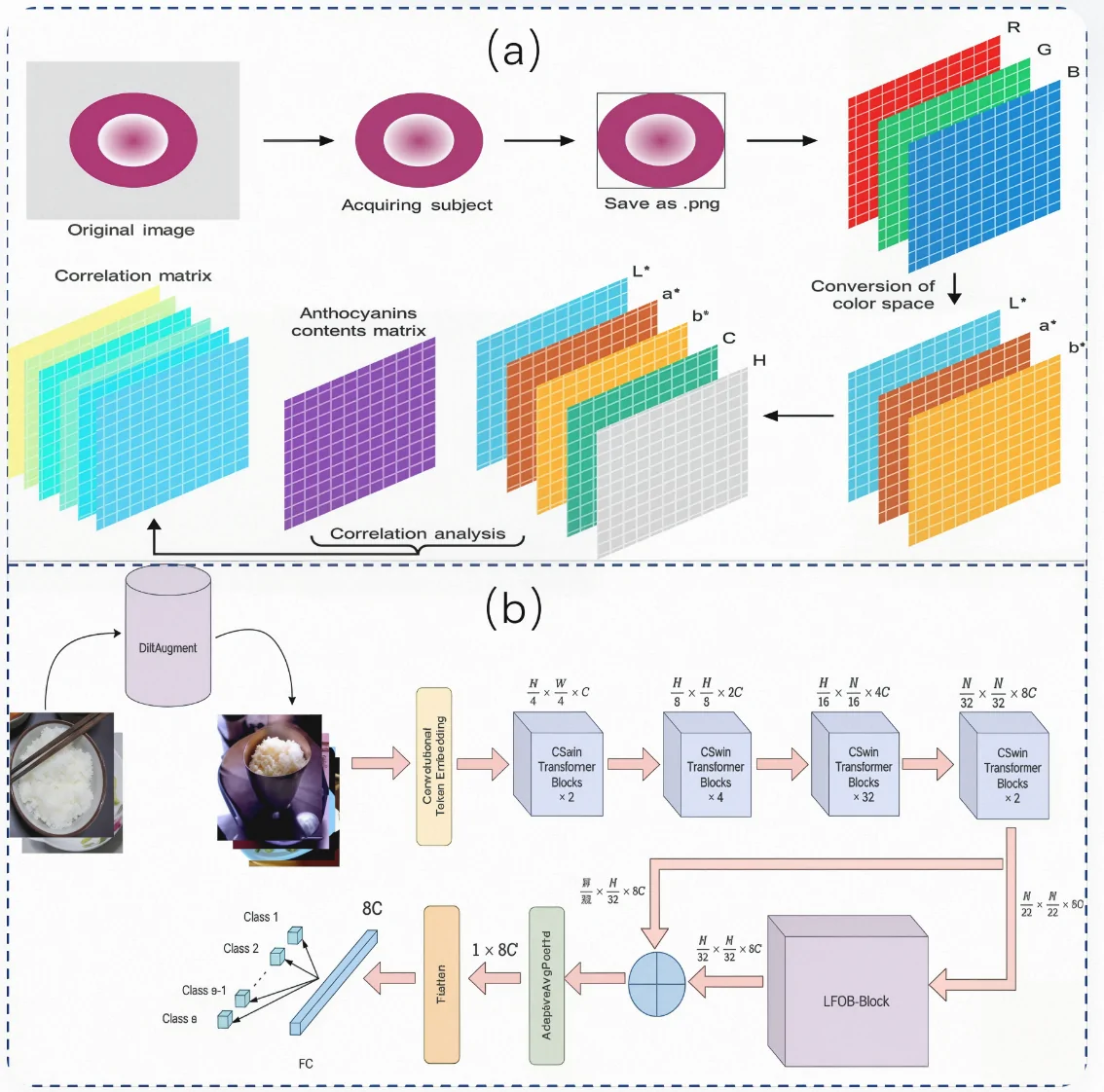
Schematic diagrams of the FoodCSWin-related model. (**a**) Process flow of image recognition and correlation analysis. The figure was reproduced from [97], with permission from Elsevier, 2024. (**b**) Architectural framework of the FoodCSWin structure. The figure was reproduced from [99], with permission from Elsevier, 2025.

Image recognition has been employed to characterize food color changes and to correlate visual information with physicochemical indicators. In the study of changes in substances in food, Li et al. developed a script based on image recognition technology to visualize the correlation matrix between the anthocyanin content and color of BSC images (Figure 5a) [97]. It was also demonstrated that there was a strong positive correlation between anthocyanins and color, mainly concentrated in the periphery of the sample, following a concentric pattern. The combination of image recognition technology and deep learning has also been widely applied in food recognition and classification tasks. You et al. employed computer vision and deep learning techniques to extract color features from dried green tea samples [98]. They developed an artificial neural network model integrating a 1D convolutional neural network, partial least squares, and a backpropagation algorithm, successfully visualizing the variations in moisture content. Xiao et al. developed the FoodCSWin model [99], which integrates the global features obtained from the CSWin Transformer with residual network connections, as illustrated in Figure 5b. The CSWin Transformer employs multi-layer image enhancement modules to extract enriched feature information. In food recognition experiments, the model was validated on the public datasets ChineseFoodNet and VireoFood172, achieving accuracies of *85.67*% and *94.11*%, respectively, on their validation sets. Such food identification models can serve a supportive role within food inspection systems, for instance by facilitating product recognition, inventory management, food portion estimation, or providing a basis for subsequent quality assessment. However, for direct application in food safety inspection, these models require further calibration and validation against independent reference data.

Image recognition technology is also widely used in the integrity inspection of food and utensils packaging. The neural network is used to learn its features, so that the extracted features represent the commodity, and finally the commodity is identified through the constructed model [100], the appearance characteristics of the packaging are analyzed, such as whether the sealing is tight, whether the packaging is damaged, etc., and the model can find the packaging defect in time, so as to ensure the safety of the product. Medus et al. integrated hyperspectral imaging (HSI) with CNN [101], and CNN is used as a classifier to apply hyperspectral imaging to carry out real-time online control classification of food packaging. In the food tray packaging experiment, the global fault detection accuracy was greater than *94*%, with a total calculation time of between *70* and *105* milliseconds. Liu et al. used the R-G grayscale image method to capture fruit images and employed block classification combined with SVM to extract color and texture features from the blocks [102]. The proposed method achieved a *FNR (false negative rate)* of *4.65*% and a *FPR (false positive rate)* of *3.50*%. CNN-based image recognition systems can more accurately analyze the external features of food and its packaging, enabling more precise detection.

A major limitation of appearance-based visual assessment methods, such as image recognition, is their high sensitivity to image acquisition variability. Factors such as illumination intensity and color temperature, shadows, specular reflections, background texture, sample orientation, camera distance and angle, image resolution, and camera type can all alter image features and consequently affect model predictions. For instance, when both training and test sets are acquired under highly standardized conditions, models may inadvertently learn cues related to the background, equipment, or acquisition procedure rather than features intrinsic to food quality itself. Therefore, studies should explicitly report imaging conditions, color calibration procedures, background treatment methods, and data augmentation strategies. More importantly, model robustness must be evaluated using images acquired under diverse lighting conditions, background environments, camera systems, and sample orientations.

### 3.2. Real-Time Detection in Dynamic Scenarios

The modern food processing industry, characterized by large-scale operations and diverse product varieties, faces one of the most formidable challenges for suppliers and producers: real-time monitoring of supply chains and comprehensive management of food quality and safety. Consequently, the development of rapid, non-destructive, and efficient detection methods has become critical to addressing these concerns [103].

**Figure 6:**
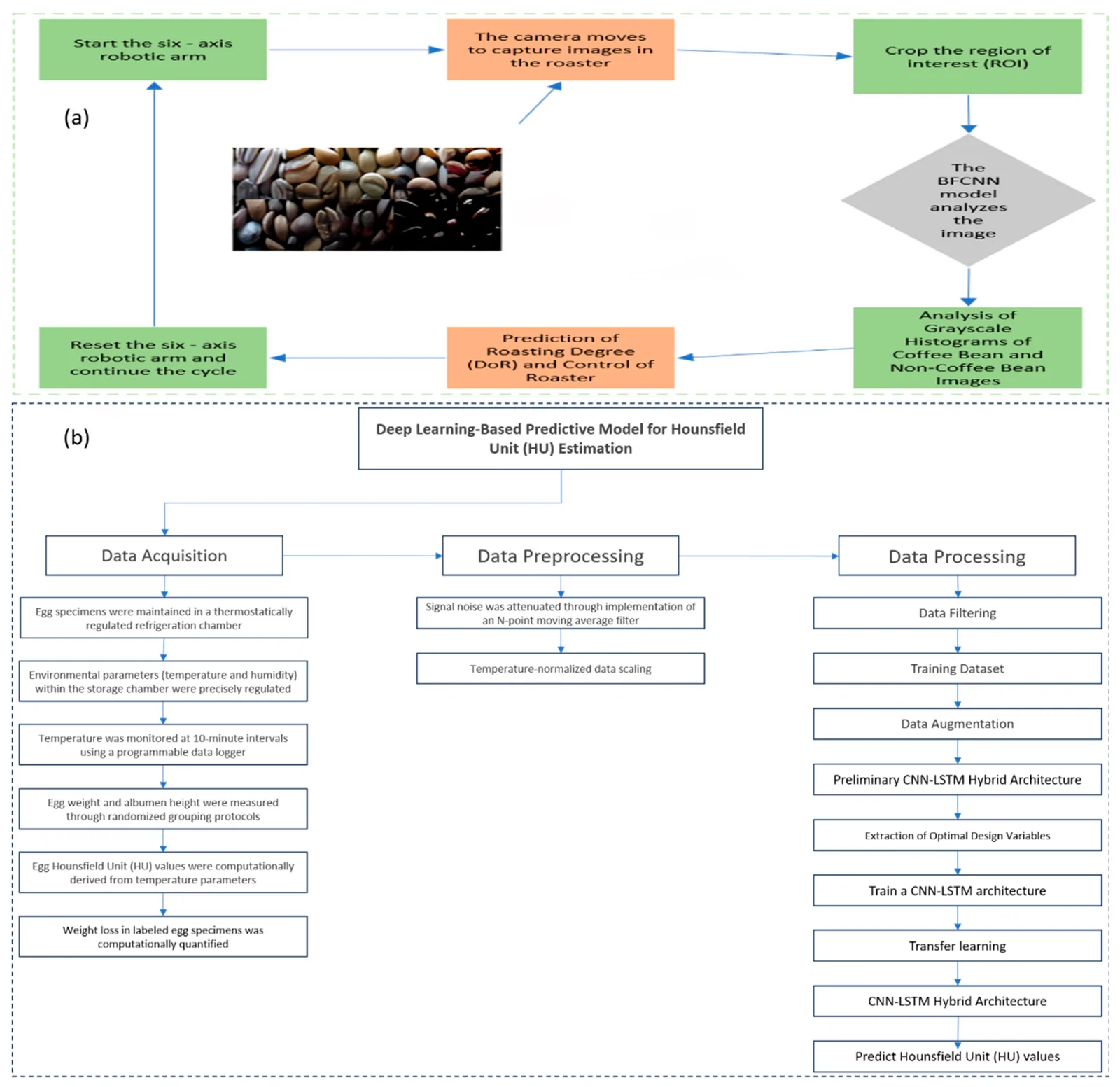
Schematic diagrams of food detection cases in dynamic scenarios using deep learning combined with imaging technology. (**a**) Schematic of a real-time coffee bean roasting degree control model using computer vision and CNN. The figure was reproduced from [105], with permission of the Creative Commons CC-BY, 2024. (**b**) Flowchart of a deep learning model combining CNN and LSTM for predicting the Hounsfield Unit (HU) of eggs in cold chain logistics. The figure was reproduced from [107], with permission of the Creative Commons CC-BY, 2022.

In the food processing production line, the camera installed on the production line collects food images in real time and uses the pretrained CNN model for rapid analysis, which can detect food quality problems in real time, such as foreign matter mixing, irregular shape, etc. Commonly used techniques, such as computer vision technologies, due to their rapid, non-destructive, consistent, and objective characteristics, have become the primary methods in food monitoring [98,104]. The intelligent food processing robotic system (FPRIS) developed by Kim and Kim integrates a 3D-printed robotic arm with CV and CNN [105]. As illustrated in Figure 6a, this system employs the CNN to classify coffee beans within a roaster and enables real-time control of the roasting process. By integrating gas and image sensor data to evaluate coffee bean quality, the FPRIS demonstrates the capability to achieve precise control over the degree of roasting (DoR), thereby optimizing roasting consistency and product outcomes. In cold chain transportation, image recognition technology can be integrated with temperature sensors, where artificial intelligence (AI) uses data collected by the sensors to predict and monitor quality changes in food cold chains [106]. As shown in Figure 6b, Kim et al. stacked CNN and LSTM algorithms together to form a deep learning model with hyperparameter optimization to monitor the freshness of eggs during transportation in real time by predicting the thermal units in cold chain transportation [107].

The key to real-time detection lies in the efficiency and accuracy of the model. In dynamic food inspection and online monitoring, the data acquisition frequency is a critical factor influencing the real-time requirements of a system. The data acquisition frequency may vary significantly across different application scenarios. For instance, visual inspection on production lines typically requires continuous image capture determined by conveyor belt speed and sample spacing, whereas cold chain monitoring may collect temperature, humidity, or gas concentration data at fixed time intervals. Real-time detection does not imply that all systems must acquire data at millisecond-level frequencies; rather, it requires that the processing and decision-making speed of the system be matched to both the rate of change of the target object and the data acquisition frequency. Higher data acquisition frequencies result in larger volumes of data to be processed per unit time, thereby imposing greater demands on model inference speed, storage capacity, data transmission bandwidth, and hardware computational resources. To enhance the real-time performance of models and enable real-time detection under high data acquisition frequencies, researchers have employed various optimization strategies, such as model simplification and hardware acceleration. For example, Chidziwisano et al. demonstrated that the MobileNet and DenseNet binary classifiers maintained significant accuracy (above *0.8*) and F1-score (above *0.8*) compared to all other models [108]. Wang et al. enhanced the YOLOv4 model by employing a lightweight YOLOv4-Tiny architecture for potted flower detection, achieving accurate and rapid identification [109]. Integrated with a ZED 2 camera for 3D point cloud extraction, the system demonstrated a mean square error of *18.1* mm in flower center localization and a maximum positioning error of *25.8* mm under varying lighting conditions. The aforementioned research confirms that employing methods such as lightweight neural network architectures can significantly improve model speed without substantially compromising performance.

However, the real-time performance of these deep learning models measured in laboratory or pilot-scale experiments may not fully represent their actual performance under industrial deployment. Food processing production lines may involve challenges, such as varying conveyor belt speeds, vibrations, motion blur, occlusions, product overlap, illumination changes, and irregular product orientations. Similarly, cold chain and warehouse environments may be affected by lighting fluctuations, unstable network connectivity, sensor drift, and variations in product batches or storage conditions. Therefore, model evaluation should not be limited to accuracy and inference time, but should also encompass throughput, latency, missed detection rate, robustness under motion and illumination variations, and performance on independent production batches.

### 3.3. The Technical Limitations of Image Recognition Technology in Food Detection Applications

Although image recognition technology has made significant progress in food quality inspection, there are still some technical limitations that limit its wide promotion in practical applications.

Above all, datasets play a vital role in intelligent systems and have a great impact on the performance of models [110]. Dataset scale and representativeness remain fundamental issues. Food quality datasets are typically constructed for specific experiments and may contain only limited samples from a single cultivar, origin, batch, or season. Rare yet critical categories—such as early spoilage, minor bruising, mold growth, contamination, and subtle packaging defects—should receive adequate attention during dataset construction. Meanwhile, small and homogeneous datasets increase the risk of overfitting and may lead to unstable model performance evaluations.

Second, class imbalance should be explicitly addressed in classification, detection, and segmentation tasks. In most food inspection datasets, the number of normal products typically far exceeds that of defective or hazardous ones. Models optimized primarily for overall accuracy may become biased toward the majority class, thereby failing to detect minority-class samples. In addition to overall accuracy, researchers should report per-class precision, recall, F1-score, confusion matrices, PR-AUC, and false negative rates for non-conventional classes. Furthermore, approaches such as class-weighted loss functions, oversampling, and targeted data augmentation may help improve the recognition of minority and high-risk categories.

In addition, the adaptability of image recognition technology in complex environments needs to be improved [111]. For example, in a scene with unstable lighting conditions or complex backgrounds, the detection accuracy of the model may be reduced. Models trained under controlled conditions may experience performance degradation when deployed across different production lines, warehouses, retail environments, or mobile devices. At the same time, real-time detection in dynamic scenarios puts forward higher requirements for the computational efficiency of models, which may be difficult for existing hardware devices to meet. Strategies such as color calibration, standardized preprocessing, background randomization, illumination augmentation, domain adaptation, and cross-device testing can help mitigate these issues.

The application of image recognition technology in food quality inspection shows great potential and innovative value. Through automatic image analysis, food appearance quality assessment and real-time detection in dynamic scenarios can be efficiently realized, which significantly improves the efficiency and accuracy of food quality inspection. Building on this foundation, the integration of deep learning with imaging technologies has been extensively applied across diverse food inspection scenarios, as summarized in Table 2. This synergistic approach demonstrates robust adaptability in addressing complex detection challenges while maintaining operational efficiency and analytical precision. However, the limitations of dataset size and quality, insufficient model interpretability, and adaptability in complex environments are still challenges for current technologies.

It should be noted that the entries listed in the second column of Table 2 do not all represent the same type of information. Some entries refer to the tested food matrix, such as turmeric, rice, beef, dairy products, oats, or yeast, whereas others refer to the target hazardous component, quality attribute, or analytical objective, such as acrylamide, foodborne pathogens, adulteration, or umami-related peptides. This difference reflects the heterogeneous objectives of the studies included in this review. The food matrix represents the material in which the analysis was conducted, while the target attribute or hazardous component represents the quality, safety, authenticity, or compositional property being evaluated.

### 3.4. Computational Efficiency and Deployment Considerations

Based on the approaches reviewed in this manuscript, the computational time for analysis and decision-making varies considerably across different model architectures and data modalities. CNN-based image recognition models typically require 50–200 ms per inference on standard hardware, with lightweight variants achieving sub-50 ms inference on edge devices. HSI-integrated models, which combine spectral processing with image classification, generally operate within 70–105 ms per sample, as reported in the literature. RNN/LSTM-based models for sequential data processing typically require 100–300 ms per inference, depending on sequence length and network depth. Multimodal fusion approaches, which process and integrate multiple data streams, generally demand 100–500 ms due to the additional computational overhead of feature extraction and fusion operations. Transformer-based architectures, while offering superior accuracy for complex tasks, typically require 200–1000+ ms owing to the computational cost of self-attention mechanisms. These time ranges are generally compatible with real-time or near-real-time food quality inspection in industrial settings, where throughput requirements typically range from 1 to 10 samples per second. However, the actual inference time depends heavily on hardware configuration (CPU vs. GPU vs. edge device), model size, input data resolution, and the number of fused modalities. For deployment on resource-constrained platforms, model compression techniques such as quantization, pruning, and knowledge distillation are recommended to meet stringent real-time requirements.

## 4. Technical Applications and Advances in Multimodal Data Fusion

### 4.1. Multimodal Fusion in Food Quality Assessment

**Figure 7:**
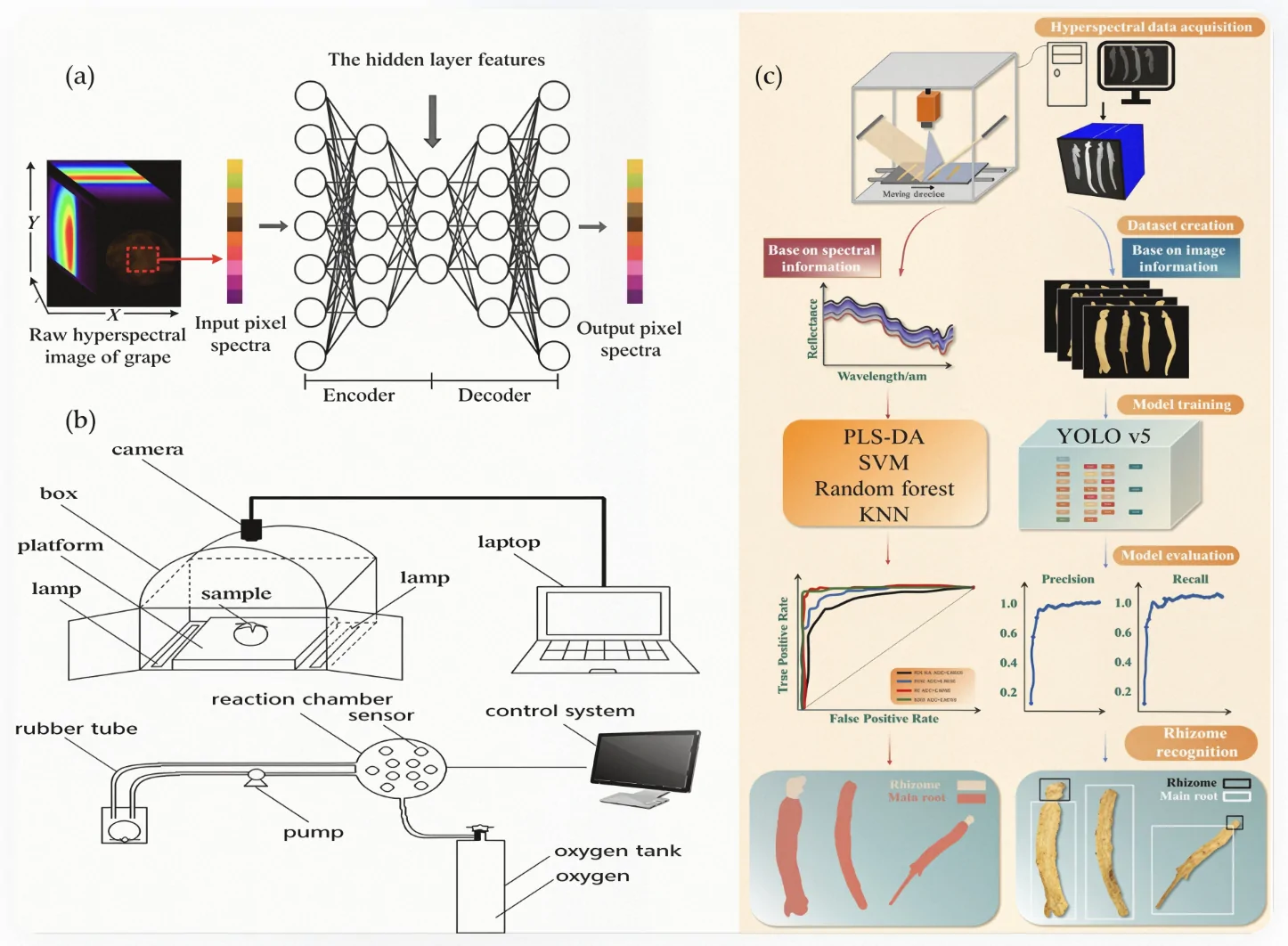
Examples of multimodal fusion in food quality detection. (**a**) The network weights obtained by training SAE based on the pixel spectra. The figure was reproduced from [11], with permission from Oxford University Press, 2023. (**b**) Quality assessment of tomatoes through integrated computer vision and electronic nose data fusion. The figure was reproduced from [125], with permission from John Wiley and Sons, 2018. (**c**) Intelligent algorithm-enhanced hyperspectral imaging system for regional quality evaluation in red ginseng. The figure was reproduced from [127], with permission from Elsevier, 2023.

Multimodal fusion technology has demonstrated substantial application value in food quality detection, particularly in the analysis and interpretation of spectral data. For example, hyperspectral imaging technology integrates image data and spectral data, enabling a more comprehensive evaluation of food freshness and safety [124]. In practical applications, researchers use intelligent models to extract image features, use hyperspectral instruments to acquire spectral images, and then combine the two through joint fusion methods, which significantly improves the detection accuracy. Xu et al. employed hyperspectral imaging technology combined with chemical methods to analyze and estimate TES and TA in grapes (Figure 7a) [11]. The team also utilized a deep-learning-based stacked autoencoder (SAE) algorithm, achieving the best prediction accuracy through the model. Huang et al. employed electronic nose and computer vision technologies (Figure 7b) and utilized fusion techniques to establish a multimodal data decision model [125]. For tomato hardness detection using a support vector machine (SVM) model, the predicted root mean square error (RMSEP) was *2.33* N. In the case of the support vector regression (SVR) model, the Rp reached *95.14*% with an RMSEP of *0.03* N. Sun et al. proposed a data fusion method based on model similarity, which analyzed and quantified the similarity relationships between feature parameters and physicochemical indicator models of fish [126]. Zhang et al. proposed a dual-scale hyperspectral imaging technology combining spectral and image information with intelligent algorithms to detect different parts of red ginseng [127], as illustrated in Figure 7c. The model takes the spectral data of red ginseng as input and outputs recognition accuracy for its distinct parts. On the red ginseng dataset, the highest accuracy, recall, and mean average precision (mAP@0.5) at an Intersection over Union (IoU) threshold of 0.5 reached *99.01*%, *98.51*%, and *99.07*%, respectively. This work provides significant implications for online and on-site quality control and authenticity identification of crude drugs or fruits.

When dealing with unconventional food samples, model predictions for high-risk targets—including foodborne pathogens, allergens, toxins, adulterants, and other analytes requiring regulatory confirmation—should be supported by validated reference methods. Integrating instrumental techniques, such as chromatography, mass spectrometry, or validated spectroscopic methods with immunochemical and molecular approaches, including ELISA, LFIA, PCR, qPCR, and LAMP, to extract multimodal feature data can enhance model accuracy in detecting unconventional samples. The most suitable application framework is a complementary workflow: a multimodal deep learning system performs rapid, non-destructive screening, while established analytical methods provide confirmatory analysis for selected or high-risk samples.

### 4.2. Multimodal Monitoring in the Food Supply Chain

Multimodal fusion technology is mainly applied in the monitoring and prediction links of the food supply chain. In the food supply chain, multimodal data fusion technology can realize the whole process of food quality monitoring. For example, by combining IoT sensor data and chemical indicator data, it is possible to monitor changes in the quality of food during transportation and storage in real time. Smart sensors connected to the Internet of Things (IoT) can perform real-time detection and collect product data during the food production, supply chain, and storage phases. Meanwhile, AI-driven models dynamically analyze these datasets, enabling real-time monitoring and continuous surveillance [128]. Morchid et al. proposed a system for fire detection in food processing that uses an embedded system to measure the amount of fire smoke in the air and the proportion of fire in the area [129], obtain data from sensors in real time, and send the machine-based Message Queuing Telemetry Transport (MQTT) protocol over the Internet to the ThingSpeak platform. In the experiment, the system successfully improved the food safety and sustainability of agriculture through accurate fire detection and fire prevention system performance.

### 4.3. Advances in Multimodal Data Fusion

Significant breakthroughs have been made in the application of multimodal data fusion technology in food quality testing. Through joint fusion methods, GNNs, dynamic fusion mechanisms, and other technologies, the detection accuracy and reliability can be significantly improved. In practical applications, multimodal data fusion technology has shown great value in food quality assessment and supply chain monitoring. In the future, with the continuous development of technologies, such as multimodal large models, self-supervised learning, and small-sample learning, multimodal data fusion will play a more important role in food quality testing. Table 3 summarizes emerging theoretical frameworks in multimodal research and their respective advantages. These methodologies are poised to serve as critical drivers for advancing multimodal data fusion in current and future applications.

## 5. Future Research Directions

### 5.1. Technical Challenges of Deep Learning in Food Quality Detection

The practical application of deep learning in food quality detection faces multi-dimensional technical challenges spanning three core aspects: data, algorithms, and implementation. These interconnected complexities significantly hinder industrial-scale deployment.

At the data level, the high acquisition costs and tedious processes for obtaining high-quality annotated data constitute a primary obstacle. Food quality detection relies on multimodal data, yet data collection may physically damage products [149,150]. Additionally, insufficient data diversity limits model generalizability. Variations in food types, production environments, and quality standards render models vulnerable to distribution shifts, leading to overfitting or biased learning—a critical issue in cross-scenario applications.

Algorithmic challenges center on model generalizability and interpretability. Although deep learning excels in complex feature extraction, its performance in food quality detection heavily depends on the representativeness of training data. Real-world scenarios demand dynamic adaptability due to food diversity, yet traditional models often suffer performance degradation when encountering new data distributions due to overfitting. Concurrently, the “black box” nature of deep learning models undermines industrial trust. In food quality detection, models must provide explicit decision logic, but existing techniques struggle to elucidate internal reasoning [76]. This limitation is particularly critical in regulatory contexts, where traceable evidence is mandated for compliance, and opaque decisions may impede adoption.

Implementation bottlenecks arise from computational constraints and data security risks. Deep learning models require high-performance computing resources, escalating deployment costs. Exponential growth in data volume and model complexity intensifies computational and energy demands [151], rendering hardware investments prohibitive for small-to-medium enterprises. Meanwhile, data security and privacy protection emerge as critical barriers. Sensitive information faces leakage or cyberattacks in open networks [152]. Resolving these challenges necessitates not only incremental innovations, but also systematic frameworks, driving cross-disciplinary collaboration to bridge the gap between laboratory validation and industrial scalability.

### 5.2. Current Practical Implementation Status of Deep-Learning-Based Technologies

Although deep learning methods have been extensively explored in food quality research, most remain at the laboratory, proof-of-concept, or prototype stage, typically demonstrating strong performance only on small datasets collected under controlled conditions. Among the techniques reviewed, RGB image analysis combined with CNNs, object detection models, lightweight networks, and edge computing systems is relatively close to practical deployment [65,153]. These approaches have shown promise in appearance grading, surface defect detection, packaging inspection, product sorting, and online process monitoring. However, their performance may be affected by illumination and background interference, class imbalance, and limited dataset scale.

Hyperspectral imaging, spectroscopic techniques, and electronic nose and electronic tongue systems, along with their integration with deep learning, can provide chemical or biological information beyond what traditional RGB images offer. However, their broader industrial adoption is constrained by instrument costs, data complexity, communication overhead, and limited external validation [154]. Models based on RNNs, LSTMs, and GRUs can analyze time-series data from cold chain and storage sensors to identify anomalous fluctuations and support quality risk prediction, but they cannot replace the underlying sensors or directly establish food safety.

Approaches based on Transformers, GNNs, GANs, and multimodal fusion are currently primarily used in research and prototype development [155]. Transformers are attractive for global context modeling and multimodal feature interaction, yet their deployment may be limited by data requirements, computational complexity, and sensitivity to domain shifts. GNNs hold promise for characterizing relationships among spectral bands, chemical indicators, supply chain nodes, and risk factors, but the generalizability of their graph construction methods across different scenarios requires further validation. GANs are particularly well suited for data augmentation and controlled image generation when authentic samples are scarce, yet generated samples cannot definitively confirm their representativeness. Multimodal fusion methods still face challenges in practical food inspection applications, as the performance of many multimodal deep learning models depends on relatively narrow domain-specific datasets and highly standardized data acquisition protocols. In real-world food safety scenarios, contamination and adulteration events typically follow a long-tail distribution, resulting in severe class imbalance and an extreme scarcity of representative, high-quality annotated samples for rare yet critical scenarios [156]. In food-safety-related applications, deep learning models should primarily serve as rapid screening or decision-support tools. Their predictions should be validated using appropriate microbiological, chemical, molecular, or instrumental reference methods before regulatory or safety-critical decisions are made.

### 5.3. Trends in Technology Convergence

#### 5.3.1. Combination of Multimodal Data and Blockchain Technology

As discussed above, advanced deep learning architectures—including Transformers, GNNs, GANs, and multimodal fusion models—have demonstrated considerable potential in food quality and safety detection, yet their practical application in the food inspection domain still requires further advancement and development. Multimodal data fusion can integrate complementary information from images, spectra, chemical indicators, and IoT sensors, thereby providing a more comprehensive assessment of food quality. However, the value of such fusion depends on the authenticity, integrity, and traceability of the underlying data. Blockchain technology offers a potential framework for meeting these requirements by providing distributed and tamper-proof records of production, inspection, storage, transportation, and transaction information. When combined with multimodal sensing and deep learning, blockchain can enhance data provenance, facilitate cross-organizational information sharing, and improve the traceability of quality-related decisions.

Blockchain lacks a widely accepted definition, but it is generally considered to be an intelligent P2P network that employs a distributed and shared database to transmit, encrypt, and record information [157]. Blockchain technology ensures the security and immutability of data through distributed ledger and encryption technology. Blockchain is a promising distributed information technology that can support the food supply chain by reducing transaction time and costs, improving traceability efficiency, and building stakeholder trust [157,158]. In food quality inspection, blockchain can be used to record multimodal data in the production, processing, transportation, and sales of food.

#### 5.3.2. Deep Integration of Artificial Intelligence and Internet of Things

The deep integration of AI and the Internet of Things (IoT) is becoming an important driving force for intelligent development. The integration of data analytics and decision-making capabilities powered by AI with real-time data acquisition enabled by the IoT not only elevates the operational efficiency of smart devices and systems but also creates transformative application opportunities across diverse fields [159]. In food quality inspection, AI combined with the IoT and blockchain plays a key role in improving inspection accuracy, increasing transparency, and addressing key challenges in food traceability [160].

For example, in smart agriculture, the combination of AI and IoT uses multi-source sensor data fusion to monitor soil, weather, and crop growth status in real time, optimize irrigation and fertilization, and use CV technology to detect crop diseases and pests. This technology not only improves the efficiency of agricultural production but also ensures the quality and safety of agricultural products. Ahmed and Shakoor proposed a viable end-to-end system architecture for carbon footprint assessment, combining IoT-enabled sensing, real-time data analytics, and predictive modeling [161]. In the field of food processing, the convergence of AI and IoT can realize the automatic control and optimization of equipment and predict failures and optimize production processes in advance by monitoring the equipment status and production environment in real time.

## 6. Conclusions and Future Prospects

### 6.1. Conclusions

Deep learning methods show great potential in food testing [162]. From image recognition to multimodal data fusion, the continuous advancement of technology provides a more efficient and accurate solution for food quality inspection. This review systematically reviews the development process, technical basis, innovative application, challenges, and future development directions of deep learning in food quality testing.

In terms of image recognition technology, deep learning models, especially CNNs, have achieved remarkable results in food appearance quality assessment and real-time detection in dynamic scenes [163]. Digital image processing (DIP) is now used for quality determination of grains, vegetables, fruits, beverages, meat, seafood, and edible oils [104,164,165,166]. Image-based CNN, YOLO, Mask R-CNN, and Transformer models have demonstrated strong performance in appearance classification, surface defect detection, packaging inspection, and object localization. Deep learning combined with hyperspectral imaging provides complementary chemical and spatial information and has been successfully applied to food composition prediction, adulteration identification, and contamination assessment. In addition, integrating images, spectra, electronic nose and electronic tongue signals, and IoT sensor data can improve the characterization of complex food quality changes compared with single-modality approaches. Lightweight models and edge computing systems are particularly promising for real-time screening in production-line and cold chain settings.

Nevertheless, the current evidence should not be interpreted as demonstrating that deep learning has achieved broad industrial maturity. Random train–test splits may overestimate model performance when images from the same batch are represented in both subsets. Class imbalance, the limited availability of defective samples, variability in inspection environments, and differences among devices and production sites also remain important barriers to reliable practical performance. External validation across batches, locations, devices, seasons, and time periods is still limited. Although data augmentation, transfer learning, and model compression may alleviate some of these limitations, further investigation is required.

The technologies currently closest to industrial implementation include computer vision systems integrated with deep learning models, such as CNNs, lightweight detection models deployed at the edge, and AIoT systems that combine computer vision with sensor data. In the short term, these technologies are more likely to support rapid screening, online sorting, process monitoring, and early warning than to fully replace confirmatory biological, chemical, and sensory analyses. Practical deployment will, therefore, likely require human–machine collaboration, together with careful consideration of hardware and maintenance costs. Data standardization, cross-domain generalization, external validation, and data security must also be addressed in subsequent research and implementation.

Overall, the central challenge has shifted from demonstrating whether deep learning can achieve high predictive accuracy to determining whether it is reliable, interpretable, and economically sustainable under real-world food inspection conditions. Future progress will depend less on continuously increasing model complexity and more on improving dataset diversity, external validation, risk-sensitive evaluation, model lightweighting, hardware efficiency, and integration with food quality management systems that are appropriate for the current maturity of emerging technologies.

### 6.2. Future Perspectives

Future research should first prioritize the development of representative, multi-source food quality datasets and standardized validation protocols. These datasets should encompass different varieties, origins, batches, seasons, storage conditions, processing stages, instruments, and acquisition environments. Model evaluation should employ batch-wise, site-wise, device-wise, and time-based external validation, and should report class-specific performance, false negative rates, calibration, uncertainty, and computational cost.

Second, lightweight architectures, pruning, quantization, and knowledge distillation should be further developed to meet the requirements of real-time industrial applications. For multimodal systems, greater attention should be paid to sensor synchronization, calibration transfer, missing-modality handling, data quality assessment, and robustness to distribution shifts. Although self-supervised learning and transfer learning may reduce annotation requirements, their effectiveness should be validated on independent target-domain datasets rather than demonstrated solely through internal validation.

Finally, explainable artificial intelligence, federated learning, blockchain-based traceability, and multimodal large models may support the development of secure and intelligent food quality systems. However, these technologies should be evaluated according to their practical contributions to detection reliability, traceability, privacy protection, and maintenance costs. The most realistic pathway toward industrial adoption is to gradually integrate laboratory-validated deep learning modules with existing imaging, spectroscopic, and sensor-based technologies that are approaching technological maturity. Through this evidence-based and risk-aware development process, deep learning can advance toward more reliable, efficient, and sustainable food quality inspection solutions for industrial applications.

## Figures and Tables

**Table 1:** Primary deep learning architectures in food quality applications.

Model Architecture	Core Principle/Mechanism	Sensing Modality	Input Data Type	Typical Food Quality Tasks	Advantages	Limitations/Challenges in Food Applications	Dataset Requirement	Computational Demand	Interpretability	Industrial Applicability
CNNs	Automatic feature extraction via convolutional layers and backpropagation.	RGB/visible images, thermal images, NIR images, hyperspectral images.	2D images, image sequences, spectral images.	Image classification, object detection.	Automated feature learning, high discriminative power.	Requires large, labeled datasets; limited generalization; high compute.	Requires a sufficiently large and representative labeled dataset.	Performance may vary depending on image resolution, spectral dimensionality, and the deployment platform.	Moderate. Feature maps, saliency maps, Grad-CAM, LIME, and SHAP can assist interpretation.	High for visual inspection and sorting, especially when lightweight architectures and model compression are available.
RNNs (LSTM)	Sequential data processing with weighted integration for prediction.	IoT sensors, temperature and humidity sensors, electronic nose/electronic tongue, sequential spectra.	Time series, spectral sequences, multivariate sensor sequences.	Risk prediction and analysis.	Handles temporal dependencies.	Gradient issues; compute-intensive; limited parallelism.	Requires temporally aligned sequential data.	Moderate to high.	Moderate to relatively high compared with deep image models, as temporal variables require inspection.	Moderate to high for process and supply chain monitoring.
Transformer	Multimodal attention via shifted windows for cross-regional feature learning.	RGB images, hyperspectral images, NIR spectra, chemical indicators, text, and multimodal sensor data.	Image patches, spectral sequences, multimodal feature tokens.	Multimodal processing, image understanding.	Long-range context capture; efficient computation.	Data-heavy; complex tuning; immature in food applications.	Typically require large-scale, diverse, or effectively pretrained datasets.	Moderate to very high, particularly for long spectra, or multiple modalities.	Moderate. Attention weights may provide useful clues.	High potential but inconsistent maturity. Industrial deployment may be constrained by data requirements, hardware conditions, and other factors.
GANs	Generate synthetic data to mimic real data.	RGB images, thermal images, hyperspectral images, and other imaging data.	Augmented samples, image-to-image mappings.	Food image synthesis and packaging defect data augmentation.	Data augmentation and synthetic data generation.	Training instability and mode collapse.	Requires a representative real-data distribution.	High during adversarial training.	Low to moderate.	As an auxiliary technology, it helps address issues of insufficient data and unstable image quality.
GNNs	Construct a graph based on tag relationships and perform graph feature learning.	Multimodal sensor networks, chemical indicator networks, knowledge graphs, relational supply chain data.	Graphs, node features, edge relationships.	Processing information of different modalities in the food industry.	Specialized in handling graph-structured data.	High computational resource consumption and insufficient generalization ability.	Requires labeled samples, a meaningful graph structure, and reliable definitions of nodes and edges.	Demand depends on graph size, edge density, number of layers.	Relatively high structurally, as node relationships and message-passing paths are inspectable.	Promising for traceability, risk analysis, and relational decision support.

**Table 2:** Applications of integrating diverse deep learning techniques with image recognition technologies in the domain of food quality and safety.

Purpose	Objective	Sensing Modality	Target Attribute/Task	Types of Deep Learning	Dataset Size	Validation Strategy	Application Results	Main Limitation	References
Food Quality Testing	Turmeric	NIR spectra + RGB images	Detection or prediction of adulteration	CNN	A total of 81 NIR spectra and 225 RGB images were collected	Internal validation	RMSEC between 0.276 and 0.965; R^2^ between 0.991 and 0.999	The available dataset is limited in size, with scarce information on adulterant types, concentration ranges, and inter-batch variability	[112]
	Rice	Hyperspectral imaging	Prediction of amylose and amylopectin contents	CNN; CNN-AdaBoost	A total of 140 rice sample spectra, comprising a calibration set of 112 samples and a test set of 28 samples	Internal validation	Rp^2^ between 0.9931 and 0.9889	Limited number of samples and possible sensitivity to cultivar, moisture, batch, and instrument variation	[113]
	Umami peptide substance	Molecular/sequence data	Prediction of umami-related peptide activity	RNN	A total of 20 standard amino acids, each encoded by a 20-dimensional 0/1 vector	Internal validation	90.5% accuracy Matthews correlation coefficient (MCC) = 0.811	This is a molecular prediction task that lacks validation through sensory evaluation or experimental approaches	[114]
	Organic food additives	Audio data	Identification of butter variations or adulteration	CNN; RNN	A total of 10,013 audio clips were created, with 90% used for model training and validation, and 10% for model testing	Random 90%/10% split; clarify whether clips from the same sample were separated at the sample level	Correctly predicted 82.66%	Audio-based features may be influenced by recording conditions, product type, and other factors	[115]
	Equine adipose-derived oil	Spectral data	Detection of horse oil adulteration	ResNet	A total of 291 horse oil samples, 100 horse oil–butter samples, 100 horse oil–sheep fat samples, and 100 horse oil–lard samples were collected, with each sample containing 591 × 3601 simulated mixture spectral data points	Internal validation	/	Simulated or prepared mixtures may not represent naturally adulterated products	[116]
	Oats	Fluorescence imaging	Detection of contaminated oats	Mask R-CNN	A total of 480 fluorescently labeled mixed oat images were collected, with 75% of the data used for training and the remaining 25% allocated to the validation and test sets	75%/25% split; clarify the proportion assigned to validation and test sets and whether splitting was image level or batch level	99.59 accuracy; 99.24 precision; 99.59 recall	Fluorescent labeling may limit direct routine application	[117]
Food Safety Testing	Common bean flour	Hyperspectral imaging	Quality detection of soybean powder	CNN; SVM	A 120 × 230-dimensional spectral matrix was obtained by averaging the spectral information extracted from each pixel to derive the mean spectrum of each image, thereby creating a representative spectrum for each bean powder sample	Internal validation	R^2^ = 0.982; RMSE = 0.165; RPD = 4.905;	Pixel-level spectra may inflate the apparent sample size	[118]
	Beef	Gas data	Classification of spoilage or quality state during storage	CNN; GRU	An electronic nose system consisting of 11 metal oxide semiconductor (MOS) gas sensors recorded the spoilage process of 12 different types of beef cuts over a period of 2220 min, with recordings taken every minute	Internal validation	0.9977 accuracy	Repeated measurements from the same samples may cause data leakage; performance should be tested across batches, cuts, storage conditions, and sensor drift	[119]
	Dairy products	Structured process or safety-risk data	Dairy food safety risk evaluation	RNN	Samples of sterilized milk from a certain brand in a province of China; the sample size was not specified in the paper	/	F1-score improved by 14.03%; execution time reduced by 41%	Small or single-source dataset; oversampling may alter class distributions	[120]
	Acrylamide	RGB images	Classification of acrylamide-positive and acrylamide-negative samples	CNN	A total of 480 potato chip images were collected, with 252 belonging to the acrylamide-negative class and 228 to the acrylamide-positive class	Internal validation	Achieved 95.09% in precision, recall, F1-score, and AUC score	Image-based prediction is indirect and may depend on processing conditions, acrylamide concentration range, lighting, and reference chemical analysis	[121]
	Yeast	Microscopic imaging	Detection of contaminated yeast	R-CNN	A total of 900 yeast colony images were collected, with microcolony images captured in PNG format (1344 × 1024 pixels, pixel size of 53)	Internal validation	mPrecision = 96.0%; mRecall = 96.3%	Morphological similarity among species and possible image-level leakage	[122]
	Foodborne pathogens	Spectra data	Detection or classification of foodborne pathogens	CNN; U-Net	A total of approximately 22,000 spectral samples were collected	Internal validation	92.77% average precision	Pure pathogen samples may not represent true independent specimens; their validity needs to be demonstrated across diverse food matrices	[123]

**Table 3:** Emerging technologies in food detection and their applications.

Method	Theory	Benefits	Role in Food Inspection	Maturity	References
Multiple Kernel Learning	Mapping original features into a higher-dimensional space to introduce non-linearity into the decision function	A useful object recognition tool where each image is represented by multiple sets of features	Integrates image, spectral, chemical, or other feature groups, particularly when modalities have different statistical properties	Established as a general machine learning fusion strategy	[130]
Graph Neural Networks	Units correspond to graph nodes, linked according to graph connections. Distinct processing units update their states and exchange information	Excel at handling complex graph structures. Primarily used for node classification, link prediction, and graph classification	Models relationships among spectral bands, chemical indicators, sensor variables, contamination factors, and supply chain nodes	Developing for food inspection	[52,131,132]
Dynamic Feature Fusion	Performing operations such as element-wise multiplication between input feature maps and generated weights to achieve dynamic feature fusion	Adaptively reweight features to improve accuracy and robustness	Adjusts the contribution of image, spectral, chemical, electronic nose, or sensor features according to sample condition and data quality	Developing	[133]
Cross-modal similarity methods	Resolve multimodal inconsistencies and model their relationships	Extract semantics from cross-modal (labeled/unlabeled) and intra-modal (same-class) pairs	Aligns image, spectral, text, chemical, and sensor representations before or during multimodal fusion	Emerging for food inspection	[134]
Multimodal Large Models	Integrate multimodal capabilities into large-scale models	Leverage large-scale datasets and extensive parameters to deliver superior solutions for multimodal tasks	Potentially supports multimodal quality assessment, inspection report generation, knowledge-assisted analysis, and decision support	Emerging for food inspection	[135,136]
Self-supervised Learning	Use a semi-automatic process to extract labels directly from the data itself	Uses unlabeled data via pseudo-labels (no human labeling)	Pretrains models using unlabeled food images, spectra, sensor streams, or multimodal records before limited-label fine-tuning	Developing	[10,137,138]
Contrastive Learning	Predict missing or unobserved data segments using known partial data	Can leverage vast amounts of unlabeled data, as generating pseudo-labels requires no manual annotation	Aligns augmented views, samples from the same food class, or corresponding measurements from different modalities	Emerging/developing	[139,140,141]
Transfer Learning	Enhance learning by establishing relationships between prior tasks and the target task	Commonly used to transfer information across related domains and establish learning/communication mechanisms between source and target tasks	Adapts pretrained image, spectral, or multimodal models to food products with limited labeled samples	Established/developing	[142,143,144]
Few-Shot Learning	Aims to achieve strong generalization performance with data-efficient architectures trained on minimal data	Enhance model generalization under limited labeled samples and address the challenges of label scarcity	Addresses rare defects, new food varieties, unusual adulterants, and limited-label inspection tasks	Emerging	[145,146]
Attention Mechanism	Dynamically shift attention to regions of critical information while suppressing irrelevant parts	It optimizes computational resource allocation under hardware constraints by prioritizing critical information processing	Selects or reweights quality-relevant features during image recognition, spectral analysis, and multimodal fusion	Established as a component; developing for multimodal fusion	[147,148]

## Data Availability

No new data were created or analyzed in this study. Data sharing is not applicable to this article.
